# Anti-Influenza Protective Efficacy of a H6 Virus-Like Particle in Chickens

**DOI:** 10.3390/vaccines8030465

**Published:** 2020-08-21

**Authors:** Wan-Zhen Zhu, Yi-Chi Wen, Shu-Yi Lin, Ting-Chih Chen, Hui-Wen Chen

**Affiliations:** Department of Veterinary Medicine, National Taiwan University, Taipei 10617, Taiwan; kitty831022@gmail.com (W.-Z.Z.); chi411chi@gmail.com (Y.-C.W.); susielin531@gmail.com (S.-Y.L.); chihchih1225@gmail.com (T.-C.C.)

**Keywords:** H6 avian influenza virus, virus-like particles, hemagglutinin, matrix 1, vaccine, adjuvant

## Abstract

H6 avian influenza viruses (AIVs) have a worldwide distribution, and they pose a potential concern for public health. In Taiwan, H6 AIVs have circulated in domestic chickens for more than 40 years, and certain strains have crossed the species barrier to infect mammals. With the goal of containing the disease, there is a pressing need to develop a safe and effective vaccine for pandemic preparedness. In this study, we prepared a virus-like particle (VLP) that consisted of the hemagglutinin (HA) and matrix protein 1 (M1) derived from a H6 AIV as a vaccine antigen, and we examined the immunogenicity and protective efficacy when combined with an adjuvant in a chicken model. Full-length HA and M1 protein genes were cloned and expressed using a baculovirus expression system, and VLPs were purified from the supernatant of insect cell cultures. We performed nanoparticle-tracking analysis and transmission electron microscopy to validate that the particle structure and properties resembled the native virions. In animal experiments, specific-pathogen-free chickens that received the H6 VLPs in combination with an adjuvant showed superior H6N1 virus-specific serum IgG and hemagglutination-inhibition antibody responses, which lasted more than 112 days. Following the H6N1 viral challenge, the vaccinated chickens showed reduced viral replication in the lungs, kidneys and conjunctival/cloacal shedding. The antibodies induced in the chickens by the vaccine were able to cross-react with the H6N1 human isolate and drifted avian H6N1 isolates. In summary, the H6 VLP vaccine elicited superb immunogenicity in vivo, and the use of an adjuvant further enhanced the antiviral protective efficacy. This vaccine formulation could potentially be used to manage H6 influenza virus infections in chickens.

## 1. Introduction

The influenza virus belongs to the family Orthomyxoviridae and has negative-sense single-stranded RNA genomes [1]. Among the four types of influenza virus, the influenza A virus has the broadest host range. Based on two proteins on the surface of the virus, hemagglutinin (HA) and neuraminidase (NA), influenza A viruses can be divided into different subtypes. To date, 18 different HA subtypes and 11 different NA subtypes have been documented [2]. H6 avian influenza viruses (AIVs) are the most commonly detected influenza viruses in wild birds and domestic poultry, and they have a broader host range than any other subtypes. An eight-year surveillance study from 1998 to 2006 indicated that H6 was the most prevalent subtype in wild birds from Europe and the Americas [3]. The infection of chickens with H6 AIV has been associated with decreased egg production, upper respiratory tract infections, morbidity, and increased mortality. Due to the mild symptoms they cause, H6 AIVs have received little attention; however, the ability of H6 AIVs to spread as well as their potential variability should not be underestimated.

Previously, the affinity to human-like receptors (α-2, 6-linked sialic acid) was found to be enhanced in the H6 AIVs circulating in poultry [4]. Certain H6 AIVs can infect mice or guinea pigs without prior adaptation [4,5]. Experimental evidence has also demonstrated that H6 AIVs can grow in the respiratory tracts of mice, ferrets and dogs, causing varied degrees of morbidity and mortality [6,7]. In Taiwan, H6N1 AIVs have been circulating for more than 40 years since the first case was reported in 1972. H6 influenza viruses were identified as potential progenitors of the highly pathogenic H5 AIV that emerged in Hong Kong in 1997 and in Taiwan in 2004 [8]. Cases of H6 AIVs infecting humans and a dog were reported in Taiwan in 2013 and 2014, respectively [9,10]. H6N1 antibodies have been detected from the poultry farmer of Taiwan [11]. These findings indicate that H6 AIVs can cross species barriers and pose a potential threat to human health.

Vaccination represents the most cost-effective way to protect against influenza diseases, and the best public health approach for its control. Conventionally, vaccine formulations consist of combinations of live attenuated viruses, inactivated virus, subunit proteins, and recombinant proteins, which elicit a specific immune response. Virus-like particles (VLPs) are one of the recombinant proteins used in vaccine formulation. This technology is a preferable method for developing vaccines because VLPs mimic the structural and antigenic properties of native viruses but lack the genetic material required for viral replication [12,13]. When presented within a host immune system, VLPs with an optimal size (20 to 200 nm) and particulate structure can be efficiently recognized by antigen-presenting cells and evoke effective immune responses without triggering the side effects [14]. VLPs can stimulate strong humoral and cellular immune responses, as they can be presented on both class I and class II MHC molecules [15]. In addition, efficient B-cell receptor crosslinking and activation are enabled by the unique repetitive surface structure of VLPs [16,17].

In light of H6 AIVs’ worldwide distribution and a broad host range, there is a pressing need to develop effective vaccines to contain the disease. In this study, we aimed to set up a vaccine platform containing an immunogenic virus-like particle (VLP) and an adjuvant. We anticipated that this combination would trigger a well-rounded immunological response for better virus protection.

## 2. Materials and Methods

### 2.1. Cell and Virus Cultures

*Spodoptera frugiperda* 9 (Sf9) and *Spodoptera frugiperda* 21 (Sf21) insect cells were used in this study. Sf9 cells were purchased from the Bioresource Collection and Research Center, Taiwan, and Sf21 cells were purchased from Gibco. The Sf9 cells were maintained in supplemented Grace’s insect medium (Gibco, Grand Island, NY, USA) containing 10% fetal bovine serum (Gibco) and 1% antibiotic–antimycotic (Gibco). The Sf21 cells were maintained in Sf-900 II SFM (Gibco) containing 1% antibiotic–antimycotic (Gibco). Both cell types were cultured at 27 °C.

H6N1 AIVs A/chicken/Taiwan/2838/2000 (2838/00) [18], A/chicken/Taiwan/3937/2012 (3937/12) and A/chicken/Taiwan/3943/2012 were isolated from chickens in Taiwan. H6 AIV A/Taiwan/2/2013 is a human isolate from the Centers for Disease Control, Taiwan [10]. For virus propagation, 200 μL of the seed virus was inoculated into the allantoic cavity of 9-day-old specific-pathogen-free (SPF) embryonated chicken eggs (JD-SPF Biotech Co., Ltd., Miaoli County, Taiwan) and incubated at 38 °C. Infective allantoic fluid (AF) was harvested at 72 h post inoculation from each embryo and clarified at 3000× *g* centrifugation for 10 min. The AF was collected and titrated to determine the 50% egg-infective dose (EID_50_)/mL, using embryonated chicken eggs as previously described [19]. For virus purification, the clarified AF was further ultra-centrifuged at 70,000× *g* for 2 h. The virus pellet was resuspended in 10 mM Tris-base, 1 mM EDTA, and 100 mM NaCl (TEN) buffer. The virus solution was then purified using a sucrose gradient solution (20%–50% in TEN buffer) and centrifuged at 50,000 rpm for 2 h. The visible virus band was collected, and the virions were pelleted using 50,000 rpm centrifugation for 2 h. The purified virions were recovered in TEN buffer.

### 2.2. Influenza Genes and the Expression Construct

Influenza HA and matrix protein 1 (M1) genes derived from A/chicken/Taiwan/3943/2012 (GenBank accession number MN088654) were synthesized by Genscript Inc. (Piscataway, NJ, USA). These two genes were cloned and ligated to the pFastBac Dual vector (Invitrogen, CA, USA). The recombinant plasmid was transformed into *E. coli* DH10Bac for gene transposition. The transposed bacmid carrying the gene of interest was used for transfection of the Sf9 cells to obtain the recombinant baculovirus (rBac-H6M1) in the culture supernatant. The rBac-H6M1 was then amplified by infecting Sf9 cells for two passages, and the virus titer was determined using a plaque assay as previously described [20].

### 2.3. Production and Purification of VLPs

Sf21 serum-free cultures were infected with rBac-H6M1 at a multiplicity of infection (MOI) of 0.1 for 7 days. The culture supernatant was harvested and centrifuged at 3000× *g* for 20 min. The VLPs were pelleted by centrifugation at 70,000× *g* for 2 h at 4 °C. The pellet was re-suspended in TEN buffer (10 mM Tris-base, 1 mM EDTA, 100 mM NaCl). The resultant solution was layered onto a sucrose gradient solution (20%–50% in TEN buffer) and centrifuged at 50,000 rpm for 2 h. Each gradient fraction was separately collected for the hemagglutination test, and the fractions demonstrating the highest hemagglutination activity and appropriate morphology under the electron microscopy were pooled as purified VLPs.

### 2.4. Sodium Dodecyl Sulfate (SDS)—Polyacrylamide Gel Electrophoresis (PAGE) and Western Blot

To determine the expression of the HA and M1 proteins, samples were mixed with 2 × sodium dodecyl sulfate (SDS)—polyacrylamide gel electrophoresis (PAGE) sample buffer (4% SDS, 20% glycerol, 120 mM Tris-HCl, 200 mM DTT, 0.02% Bromophenol blue). SDS-PAGE was conducted in 12% stain-free TGX-gels (Bio-Rad, Richmond, CA, USA) and then transferred onto a nitrocellulose membrane (PerkinElmer, Akron, OH, USA). After blocking with 5% skim milk (Difco, BD Biosciences San Jose, CA, USA), a Western blot was conducted with chicken H6N1 antiserum (1:500) as the primary antibody [18] overnight, and the goat anti-chicken IgG HRP conjugate (1:2000) (Jackson ImmunoResearch, West Grove, PA, USA) as the secondary antibody for 1 h. The membranes were developed using ECL reagents (Bio-Rad) and imaged on the ChemiDoc XRS+ system (Bio-Rad, Hercules, CA, USA). The HA content of a given VLP was analyzed by comparing the signals generated from the blots with those of the recombinant H6 HA protein (Sino Biological, #11723-V08H, Beijing, China).

### 2.5. Transmission Electronic Microscopy (TEM) and Immunogold Staining

Purified VLPs were adsorbed onto a plasma-discharged copper grid three times for 15 s each. After washing with PBS and blocking with 1% BSA, the grid was incubated with H6/HA monoclonal antibody EB2-E5 [21] (1:100) for 1 h, followed by incubation with 6 nm gold-conjugated goat anti-mouse IgG (1:20) (Jackson ImmunoResearch) for 1 h. After washing with PBS, 2% phosphotungstic acid was applied for negative staining. The particles were observed under a transmission electron microscope (JEOL JEM-1400).

### 2.6. Hemagglutination Test and Hemagglutination-Inhibition Test

Hemagglutination and hemagglutination-inhibition (HI) tests were conducted according to the OIE Terrestrial Manual 2015 protocol [22] and as previously described [20]. Briefly, 25 μL of 1 × PBS was dispensed to each well of a V-bottomed 96-well plate and 25 μL of virus suspension was placed in the first well, followed by pipetting 15 times to mix well; next, 25 μL was transferred to the next well to perform two-fold dilutions across the plate. An amount of 25 μL of 1% chicken red blood cell (cRBC) solution was dispensed to each well. The solution was mixed by gently tapping the plate. After 40 min at room temperature, the plate was tilted to observe the results.

Positive results formed a diffuse film and negative results appeared as a tear-shaped streaming of the cRBCs in the V-bottomed plates. The viral hemagglutination titer was read to the highest dilution that produced a positive reading; this end-point represented 1 hemagglutination unit (HAU). For the HI test, 25 μL of PBS was dispensed into each well of the V-bottomed plates. The serum sample (25 μL) was placed into the first well of the plate, followed by pipetting 15 times to mix well; next, 25 μL was transferred to the next well to perform two-fold dilutions across the plate. We incubated 4 HAU viral antigens with sera samples for 30 min at room temperature. After 30 min, 25 μL of 1% cRBCs was added to each well. The plate was tapped to mix well. After 40 min at room temperature, the plate was tilted to observe the results. Only those wells in which the cRBCs streamed at the same rate as the control wells were considered to show inhibition. The HI titers were indicated with respect to the highest serum dilution that inhibited virus-induced hemagglutination.

### 2.7. Chicken Immunization and Viral Challenge Studies

Groups of chickens were subcutaneously immunized twice with 432 HAU H6 VLPs only or in combination with Montanide ISA 71 VG (Seppic, Paris, France), with an interval of 21 days. Chickens receiving PBS served as a control group. Serum samples were collected before immunization and at 21, 35, 56, 77, 99, and 112 days post immunization (dpi), and the chickens were sacrificed at 112 dpi. For each group, three to five chickens were intranasally challenged with 10^6^ or 10^9^ EID_50_ (in 0.2 mL) of H6 AIV (3937/12) 3 weeks after the booster. After the viral challenge, the chickens were monitored for clinical signs, and samples, including tears and cloacal swabs, were collected. Upon sacrifice at 4 days post challenge (dpc), the lungs and kidneys of the chickens were collected for virus analysis. The chicken studies were conducted at the National Taiwan University, under an approved Institutional Animal Care and Use Committee protocol (IACUC approval no. NTU104-EL-20). All animal experiments were carried out in accordance with the approved guidelines.

### 2.8. Enzyme-Linked Immunosorbent Assay (ELISA)

We evaluated the antigen-specific antibodies in the chicken sera in 96-well flat-bottomed plates (Nunc) coated with 100 ng/well H6N1 virions (3937/12) overnight at room temperature, followed by blocking with 5% skim milk. The test chicken sera were serially diluted and incubated for 1 h. Following the washes, 100 μL of goat anti-chicken IgG HRP conjugate (Jakson ImmunoResearch, West Grove, PA, USA) diluted 1:2000 in blocking buffer was added to each well, and the wells were incubated for another 1 h. After an additional three washes, the wells were incubated with 100 μL of KPL SureBlue Reserve TMB Microwell Peroxidase Substrate (SeraCare, Milford, MA, USA), and the color was allowed to develop in the dark for 10 min. A quantity of 100 μL of KPL TMB stop solution (SeraCare) was added to stop the reaction. Finally, the OD at 450 nm was detected using an automated plate reader (Synergy H1, BioTek, Winooski, VT, USA).

### 2.9. Viral RNA Extraction, Reverse Transcription, and Quantitative PCR (qPCR)

We extracted the viral RNA using the Viral Nucleic Acid Extraction Kit II (Geneaid, Taipei, Taiwan), following the manufacturer’s protocol. cDNA was generated using the QuantiNova Reverse Transcription Kit (Qiagen, Valencia, CA, USA). For qPCR, the reaction mixtures were prepared using PrimeTime^®^ Gene Expression Master Mix (Integrated DNA Technologies, Bothell, WA, USA) according to the manufacturer’s instructions with virus-specific primers and the probe [23]. The qPCR reactions were performed as previously described [24,25].

### 2.10. Virus Re-Isolation in Embryo Eggs

Chicken cloacal swabs were immersed in 0.5 mL of the virus medium, and the lungs and kidneys were homogenized in 3 mL of virus medium. The homogenized samples were clarified for virus re-isolation in SPF chicken embryos. We determined the virus positivity by inoculating 0.L ml of each swab homogenate or 0.2 mL of the organ homogenate into two embryos. After incubation for 7 days, the embryos were opened and the hemagglutination activity of the AF was examined.

### 2.11. Statistical Analyses

The data were analyzed by ANOVA followed by Dunnett’s or Tukey’s multiple-comparison tests using Prism 6 (GraphPad, San Diego, CA, USA). *p*-values smaller than 0.05 were considered significant.

## 3. Results

### 3.1. Production and Purification of H6 VLPs

**Figure 1:**
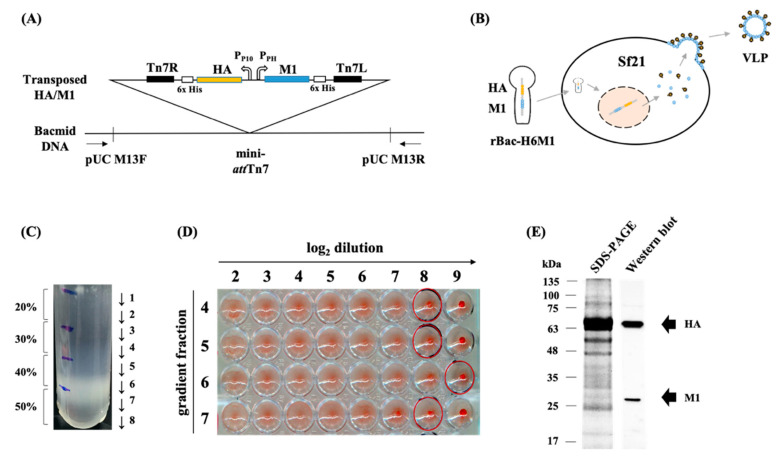
Generation and purification. A schematic diagram of H6 virus-like particle (VLP) construction and expression. (**A**) A schematic diagram of H6 VLP construction and expression. The recombinant plasmid carrying the hemagglutinin (HA) and matrix protein 1 (M1) genes derived from the H6 avian influenza virus (AIV) was transposed onto the bacmid DNA. (**B**) VLPs that assembled HA and M1 proteins were harvested in the rBac-H6M1-infected Sf21 culture supernatant. (**C**) The H6 VLPs were purified through sucrose density gradient centrifugation. (**D**) The hemagglutination activity of purified VLPs was verified with the HA test. (**E**) The expression of the HA and M1 proteins on the VLPs was analyzed using SDS-PAGE and validated by Western blot using the antiserum directed against H6 AIVs.

The full-length HA (1713 nt) and M1 (789 nt) genes of A/chicken/Taiwan/3943/2012 were cloned into the pFastBac Dual vector (Figure S1) and transposed onto the bacmid DNA (Figure 1A). The Sf21 cells were infected by the recombinant baculovirus, rBac-H6M1, to produce VLPs in the culture supernatant (Figure 1B). The titer of the rBac-H6M1 was determined to be 1.4 × 10^6^ plaque-forming unit/mL using plaque assays. Based on the optimization results, all VLPs in the remainder of the study were prepared using Sf21 cells infected with the rBac-H6M1 with an MOI of 0.1. The production of VLPs was first analyzed by SDS-PAGE and Western blotting, and then the VLPs were purified using the sucrose gradient centrifugation. The fractions in 40–50 wt.% sucrose demonstrated a visible banding (Figure 1C), indicating the presence of concentrated VLPs. Further examination revealed that fractions 4, 5, 6, and 7 showed high HA activity up to 512 HAU per 25 μL (Figure 1D). Simultaneous expression of HA (66 kDa) and M1 (27 kDa) was shown in the SDS-PAGE, followed by validation by Western blot as shown in Figure 1E.

### 3.2. Characterization of H6 VLPs

**Figure 2:**
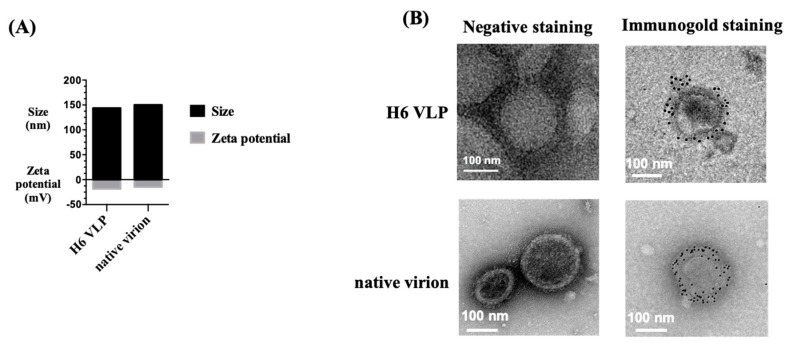
Characterization of H6 VLPs. (**A**) The size and surface zeta potential of H6 VLPs and native virions were measured using NanoSight. (**B**) The H6 VLPs and native virions were imaged using TEM under negative staining and immunogold staining using the H6/HA monoclonal antibody. Bar = 100 nm.

To examine the formation and antigen display of the VLPs, we performed nanoparticle-tracking analysis and immunogold staining. According to the NanoSight analysis, the average size of the VLPs was 144 nm, similar to that of the native virions (150 nm), and the surface potential of VLPs was −18.5 mV, compared to that of native virions at −14.7 mV (Figure 2A). Under the negative staining in the TEM observation, unimodal particles were observed, confirming successful preparation of VLPs with morphology that resembled that of the native virions. HA antigen expression on the surface of the particles was validated via immunogold labeling, mirroring the staining pattern on the native virions (Figure 2B). In addition, the HA protein content ratio on the VLP was analyzed using Western blotting and compared to the H6/HA recombinant protein using the same total protein amount (5 μg). The results showed that in the current VLP formulation, the HA protein contributed approximately 8% of the total protein of the VLPs (0.4 μg HA in 5 μg VLPs) (Figure S2).

### 3.3. Immunogenicity of H6 VLPs with ISA 71 VG

**Figure 3:**
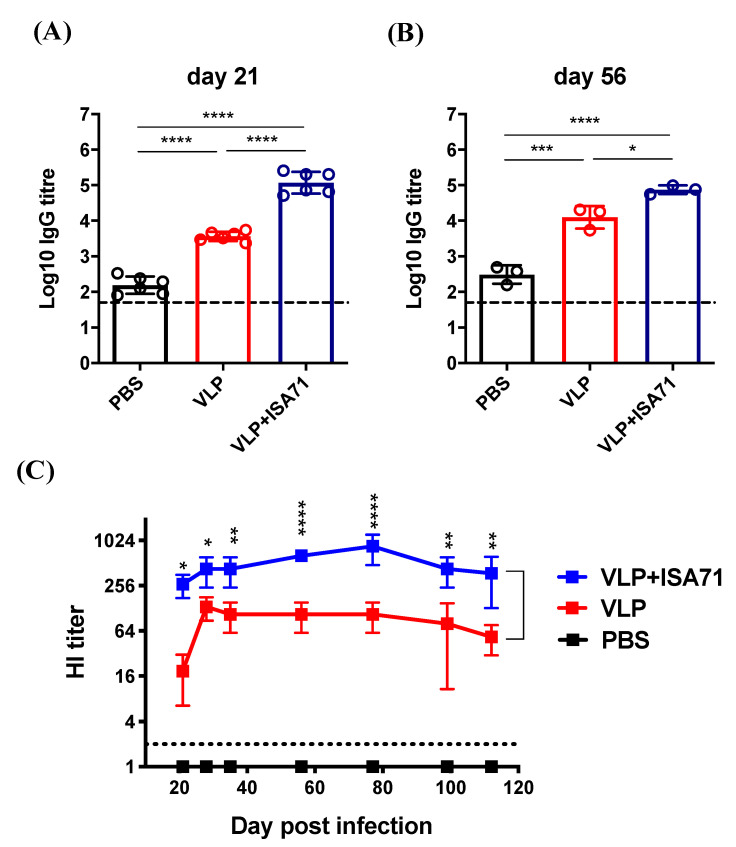
The immunogenicity of H6 VLPs with ISA 71 VG in a chicken model. (**A**) Specific-pathogen-free (SPF) chickens were prime-boost vaccinated on Days 0 and 21 with PBS, VLPs alone, or VLPs with commercial adjuvant ISA 71 VG, and blood samples were collected. H6N1 AIV-specific IgG titers were analyzed by ELISA on day 21. (**B**) H6N1 AIV-specific IgG titers were analyzed by ELISA on day 56. (**C**) H6N1 AIV-specific HI titers were analyzed by a HI test. Error bars represent the means ± S.D. The horizontal dotted lines mark the detection limit for the antibody titer. Serum IgG titers among groups were compared using one-way ANOVA followed by Dunnett’s multiple-comparison test. The HI titers among vaccination groups and the control group were compared using two-way ANOVA followed by Tukey’s multiple-comparison test, and the differences between the VLP alone and ISA 71 VG-adjuvanted groups are shown (* *p* < 0.05; ** *p* < 0.01; *** *p* < 0.001; **** *p* < 0.0001).

To examine the immune-potentiating effect of the H6 VLPs in chickens, 3-week-old SPF chickens were administered a primary and a booster vaccination of PBS or 432 HAU H6 VLPs with or without adjuvant ISA 71 VG on day 0 and day 21. The capacity of these groups to display a humoral immune response was evaluated by detecting the presence of H6N1 AIV-specific antibodies in the sera of the vaccinated chickens using ELISA or a HI test. We analyzed the time-course of HA-specific antibodies induced by the vaccine group for 112 days. As shown in Figure 3A, on day 21, a single-dose inoculation of VLPs in chickens with or without the adjuvant effectively elicited anti-H6N1 AIV antibodies, with a mean titer of 10^3.6^ in the VLP alone group and 10^5.1^ in the ISA 71 VG-adjuvanted group. The ISA 71 VG-adjuvanted VLPs induced a significantly higher antibody response than the VLPs alone (*p* < 0.0001).

After the booster immunization, both the VLP alone and adjuvanted groups continued to show high titers. At 56 dpi, the mean titer in the VLP alone group reached 10^4.1^, and the titer in the ISA 71 VG-adjuvanted group was 10^4.9^ (Figure 3B). For the HI titer, over all evaluated time-points, the ISA 71 VG-adjuvanted group showed 2- to 16-fold higher titers than the VLP alone group. The titers in the ISA 71 VG-adjuvanted group peaked at 77 dpi (mean titer: 853); though they slightly declined afterward, the titer was still sustained through 112 dpi (mean titer: 373). Overall, the ISA 71 VG-adjuvanted VLP vaccine induced a high and durable HI antibody response in chickens (Figure 3C).

**Figure 4:**
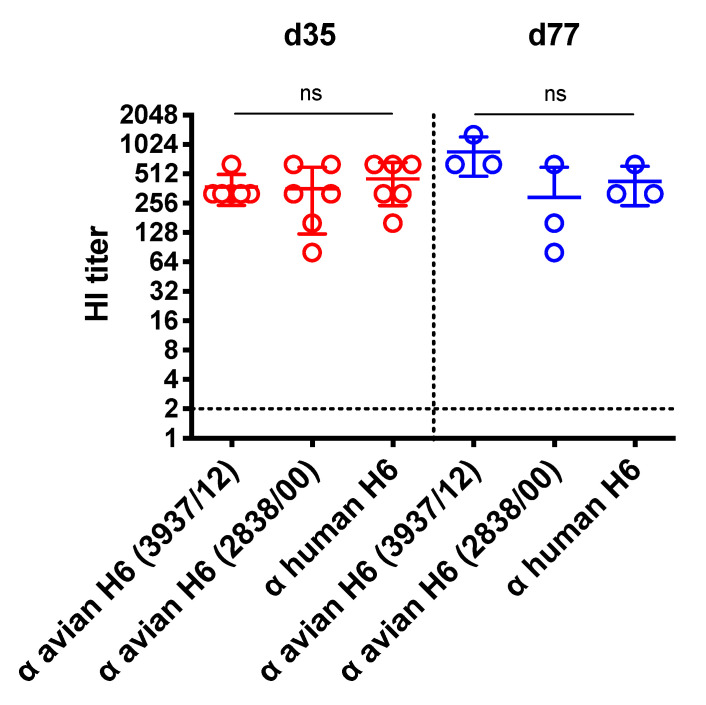
HI titers against the H6N1 human isolate and drifted avian H6N1 isolates. Cross-reactive antibody titers against the H6N1 human isolate and drifted avian H6N1 isolates from the VLP plus ISA 71 VG adjuvant vaccinated chickens were analyzed at 35 days post immunization (dpi) and 77 dpi using a HI test. Error bars represent the means ± S.D. The horizontal dotted lines mark the detection limit for the antibody titer. The horizontal dotted line marks the detection limit for viral copies. Titers among groups were compared by one-way ANOVA followed by Tukey’s multiple-comparison test. ns: non-significant.

The cross-reactivity of the VLP plus ISA 71 VG-induced serum antibodies against other avian or human H6N1 isolates was examined by a HI test. As shown in Figure 4, the mean HI titers were 373, 360 and 453 at 35 dpi against chicken H6N1 3937/12, 2838/00 and human H6N1 (A/Taiwan/2/2013), respectively. At 77 dpi, while the VLP plus ISA 71 VG-induced serum consistently showed a high antibody level (mean titer: 853) against the 3937/12, the cross-reactive HI titers against the 2838/00 or the human H6N1 slightly decreased. The HI titer against the chicken H6N1 2838/00 was 293; toward the human H6N1, it was 426. There were no significant differences among these groups.

### 3.4. Protective Efficacy of H6 VLP Vaccine

**Figure 5:**
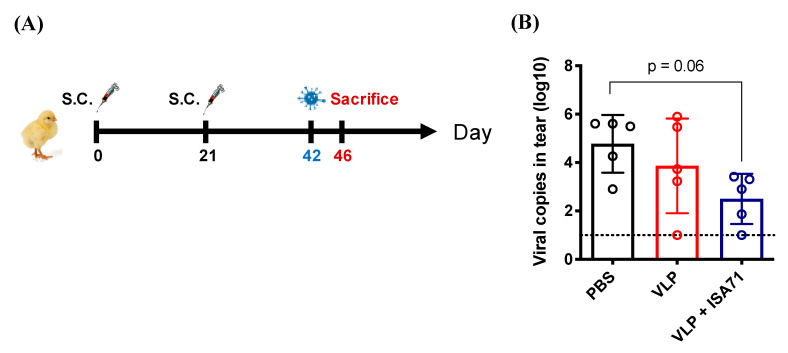
The protective efficacy of H6 VLPs in a chicken model. (**A**) The vaccine immunization and virus challenge schedule in chickens. Three weeks after the booster immunization, chickens were intranasally challenged with 10^6^ 50% egg-infective dose (EID_50_) H6 AIV. (**B**) Viral RNA in tears at 4 dpc was analyzed by RT-qPCR. Error bars represent the means ± S.D. The horizontal dotted line marks the detection limit for viral copies. Viral copies among groups were compared by one-way ANOVA followed by Tukey’s multiple-comparison test.

To examine the protective efficacy of the H6 VLP vaccines in chickens, a H6 AIV 3937/12 viral challenge (10^6^ EID_50_ per chicken) was conducted 21 days after the two subcutaneous injections. After challenge, the clinical signs of chickens were observed on a daily basis, and virus shedding and the tissue viral load at 4 dpc were determined using qPCR and virus re-isolation (Figure 5A). Since two days following challenge, the PBS-receiving chickens showed 100% (5/5) morbidity, where mild tracheal rales and nasal-ocular discharge were recorded. In contrast, 40% (2/5) of the VLP alone group showed aforementioned signs, and 0% (0/5) of the ISA 71 VG adjuvated group developed clinical signs.

For the conjunctival shedding (Figure 5B), the mean number of viral copies in the tear samples of PBS-receiving chickens was 10^4.7^, the highest among the five groups. Both VLP-vaccinated groups of chickens demonstrated lower levels of viral shedding in their tears (VLP alone: 10^3.8^; VLP plus ISA 71 VG: 10^2.5^). Although statistical significance was not reached (*p* = 0.06), the viral copy number from the VLP plus ISA 71 VG group showed the lowest viral shedding among three groups. For the cloacal shedding, as indicated in Table 1, 80% (4/5) of the chickens in the PBS-receiving group shed virus through the cloaca, whereas 20% (1/5) in the VLP alone group and 0% (0/5) in the ISA 71 VG-adjuvant group showed re-isolated virus in the cloacal swabs. Additionally, while 80% (4/5) of the chickens in the PBS-receiving group demonstrated viral replication in the kidney, none of chickens from the two VLP-vaccinated groups had live virus detected in this organ. Similarly, in the lung samples, 100% (5/5) of the chickens in the PBS-receiving group demonstrated viral replication in the lungs, whereas 40% (2/5) in the VLP alone group and 0% (0/5) in the ISA 71 VG-adjuvant group showed re-isolated virus in the lung.

Furthermore, a different dose of challenge (10^9^ EID_50_ per chicken) was also used to validate the protective efficacy of VLP plus ISA 71 VG under a higher amount of viral challenge. The results showed that 100% (3/3) of PBS-receiving chickens developed notable clinical signs, including tracheal rales, nasal-ocular discharge, head shaking and watery feces, whereas 0% (0/3) of VLP plus ISA 71 VG group showed symptoms. Nevertheless, due to the low pathogenic nature of H6N1 AIVs, there was no mortality observed even under the high challenge dose. For the virus re-isolation in the lungs, as indicated in Table 1, 100% (3/3) of the PBS-receiving chickens demonstrated viral replication, whereas 0% (0/3) in the VLP plus ISA 71 VG adjuvant group had re-isolated virus. These results support the notion that the VLPs produced in this study elicited desirable anti-H6 AIV protection in chickens.

## 4. Discussion

Avian influenza VLPs have been generated in various expression systems, including mammalian [26], insect [20,27] and plant cells [28], and they harbor different glycosylation pathways such as the extent of N-glycan branching and elongation [29]. A previous study reported the vaccination efficacy of a 293T cell-derived H6 VLP carrying the viral HA, NA, M1, and matrix 2 (M2) proteins in a mouse model [30]. Here, using an avian model, we demonstrated that an insect cell-derived H6 VLP with HA and M1 proteins is sufficient to confer protective immunity against a live viral challenge. HA protein is the major surface glycoprotein of influenza viruses. HA can recognize target cells by binding to their sialic-acid-containing receptors and mediating fusion between the viral and the endosomal membranes after endocytosis [31]. On the other hand, M1 is a central component of the virus particle, forming a matrix underneath the lipid bilayer of the viral envelope and playing essential roles throughout the virus life cycle [32,33]. In this study, VLP antigenicity was validated via Western blotting using chicken antiserum. The hemagglutination activity of VLPs further confirmed that the HA proteins anchored on VLPs retained their functional stability and cRBC-binding activity. We demonstrated that vaccination with H6 VLPs combined with a commercial adjuvant induced significantly higher antigen-specific antibody titer than in other groups after single dose vaccination (measured on day 21), and a long-lasting HI antibody response was observed up to 4 months postvaccination. Following the virus challenge, viral shedding and replication in the lungs and kidneys were completely abolished in the vaccinated chickens.

Montanide ISA 71 VG was selected as an adjuvant in conjunction with VLPs to increase the potency and effectiveness of vaccination. ISA 71 VG is a commercial water-in-oil emulsion adjuvant, and has demonstrated efficacy and safety in poultry. In addition, ISA 71 VG was shown to stimulate both humoral and cellular immune responses [34]. Although not examined, the cellular immunity may also play a role in eliminating the virus in chickens receiving the VLP plus adjuvant vaccination as HA and M1 proteins have been reported to carry T cell epitopes [35]. While this study confirmed that VLP vaccination was able to protect against the homologous strain, the protection against heterologous subtypes of influenza viruses remains unknown. To improve the cross-protective efficacy against heterosubtypic AIVs, various strategies have been adopted in previous studies. Pushko et al. prepared H5/H7/H9/N1/gag VLPs containing HA antigens derived from several subtypes of AIVs [36]. Sunwoo et al. constructed a new HA containing HA heads of different influenza subtypes grafted onto a conserved HA stalk, and used them to assemble VLPs [37]. In another study, Steel et al. incorporated headless HA into VLPs, and vaccinated mice with the headless HA VLPs or the full-length HA VLPs. Mice vaccinated with the headless HA showed greater activity against heterologous strains than those vaccinated with the full-length HA [38]. Other studies also have employed the consensus region of the HA stem or conserved influenza M2 epitopes in their vaccine design [39,40], demonstrating that a variety of VLP formulations are able to elicit cross-protective immunity against homologous and heterologous strains. In the future, H6 VLP from this study may be modified by further incorporating conserved antigenic regions that include the HA stalk domain, the nucleoprotein or ectodomain of M2 protein to provide a better heterologous protection. The lack of efficacy comparisons with the inactivated vaccine is a limitation of this study. Based on previous studies, an H9N2 VLP plus ISA 70 adjuvant conferred equal protection to the inactivated vaccine [41], and a plant-derived H6N2 VLP vaccine elicited a comparable immune response and reduced virus shedding than a commercial inactivated H6N2 vaccine [28].

## 5. Conclusions

In summary, we successfully constructed and characterized H6 influenza virus-like particles. In our in vivo study, the vaccinated chickens showed a superior and long-lasting antibody response in their serum IgG and hemagglutination-inhibition antibody titers. After the viral challenge, the vaccinated chickens demonstrated reduced viral replication in the lungs, kidneys and conjunctival/cloacal shedding. These data support our VLP as a vaccine candidate to manage influenza H6 virus infections in chickens. The VLP vaccination offers a safe and novel means to enhance the immune response.

## Figures and Tables

**Table 1:** Virus re-isolation from the cloacal swabs, lungs, and kidneys of vaccinated chickens.

Vaccination Group	10^6^ EID_50_ Challenge			10^9^ EID_50_ Challenge
	Cloacal Swab	Lung	Kidney	Lung
PBS	4/5	5/5	4/5	3/3
VLP	1/5	2/5	0/5	n/a ^1^
VLP + ISA 71 VG	0/5	0/5	0/5	0/3

^1^ n/a: data not available.

## Supplementary Materials

The following are available online at https://www.mdpi.com/2076-393X/8/3/465/s1, Figure S1: Construction of recombinant plasmid containing the full HA and M1 protein genes. Figure S2: Quantification of HA antigen in H6 VLPs.
