# Supplementary material for: Anti-Influenza Protective Efficacy of a H6 Virus-Like Particle in Chickens
Source: Vaccines (Basel). 2020 Aug 21;8(3):465. doi: 10.3390/vaccines8030465 (PMC7565593; doi:10.3390/vaccines8030465)
Supplement: Supplementary file 1 [file vaccines-08-00465-s001.pdf]

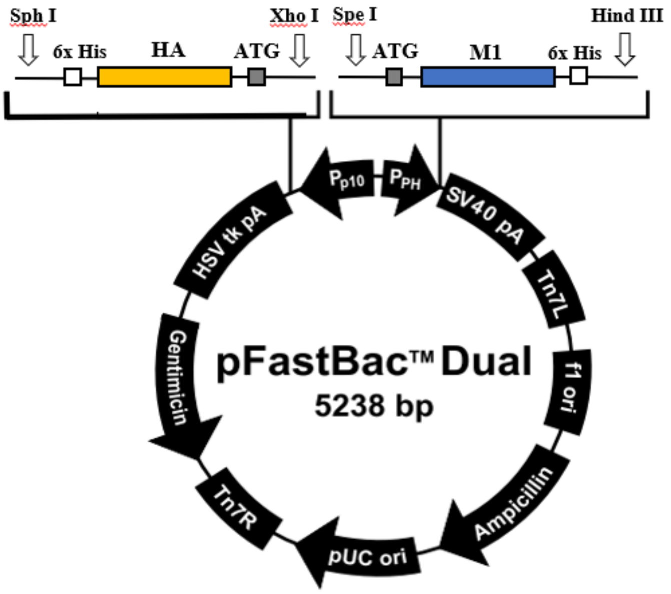

Figure S1. Construction of recombinant plasmid containing the full HA and M1 protein genes. HA and M1 genes derived from the H6 AIV from A/chicken/Taiwan/3943/2012 were cloned into the pFastBac Dual vector.

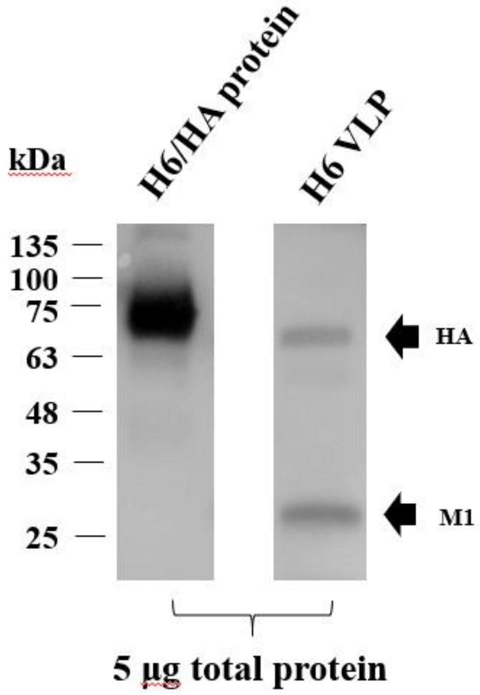

Figure S2. Quantification of HA antigen in H6 VLPs. Equal amount (5 ug) of H6/HA recombinant protein and H6 VLPs were analyzed by Western blot using chicken H6 AIV antiserum.
